# FGF2 Attenuated Inflammation-Mediated Cardiac Damage: A Novel Mechanistic Insight into the AMPK–FUNDC1–Mitophagy

**DOI:** 10.34133/research.1013

**Published:** 2025-11-24

**Authors:** Pingjun Zhu, Xi Wang, Xinjie Han, Yan Wang, Yongkai Ding, Min Zhu, Xin Zhang, Qian Xu, Yukun Li, Zhongxuan Li, Zhi Lu, Qi Zhang, Yan Yin, Guogang Xu, Yingzhen Du

**Affiliations:** ^1^ The Second Medical Center & National Clinical Research Center for Geriatric Diseases, Chinese PLA General Hospital, Beijing 100853, China.; ^2^ Chinese PLA General Hospital, Medical School of Chinese PLA, Beijing 100853, China.; ^3^ Department of Emergency, Beijing Tsinghua Changgung Hospital, Tsinghua University, Beijing 102218, China.; ^4^ Department of Cardiology, Beijing Anzhen Hospital, Capital Medical University, Beijing, China.; ^5^ Senior Department of Cardiology, The Sixth Medical Center of PLA General Hospital, Beijing 100048, China.; ^6^ Department of Automation & Institute for Brain and Cognitive Sciences, Tsinghua University, Beijing 100084, China.; ^7^ Department of Pulmonary and Critical Care Medicine, First Affiliated Hospital of China Medical University, Shenyang 110001, China.; ^8^ Health Management Institute, The Second Medical Center, Chinese PLA General Hospital, Beijing 100853, China.

## Abstract

Septic cardiomyopathy, a severe complication of sepsis, is characterized by high morbidity and mortality rates, and its effective management remains an important challenge. Although fibroblast growth factor 2 (FGF2) has been shown to exert cardioprotective effects, its role in septic cardiomyopathy has not been extensively investigated. To address this knowledge gap, FGF2 knockout (FGF2^−/−^) mice were injected with lipopolysaccharide (LPS) to establish septic cardiomyopathy in vivo, and the resulting cardiac injury was evaluated after 72 h. The results demonstrated that LPS inhibited FGF2 expression in cardiomyocytes, and genetic ablation of FGF2 exacerbated myocardial inflammation, oxidative stress, apoptosis, and cardiac dysfunction. Notably, treatment with recombinant FGF2 (rFGF2) effectively reversed these detrimental effects. Proteomic analysis revealed that FGF2 significantly modulated mitophagy, and further verification assays confirmed that FGF2 prevented LPS-induced mitochondrial injury and followed apoptosis by activating FUNDC1-mediated mitophagy. Molecular studies demonstrated that rFGF2 triggered the adenosine monophosphate-activated protein kinase (AMPK) signaling pathway, leading to the activation of FUNDC1-mediated mitophagy, which in turn mitigated myocardial mitochondrial injury and apoptosis. These findings suggest that FGF2 exerts its cardioprotective effects in septic cardiomyopathy by activating the AMPK–FUNDC1-mediated mitophagy pathway, thereby providing a potential therapeutic strategy for mitigating sepsis-induced cardiac damage.

## Introduction

Sepsis is a complex systemic inflammatory response syndrome triggered by a diverse array of endogenous and exogenous pathogens, often resulting in multiple life-threatening organ failures [1]. Notably, sepsis-induced myocardial dysfunction occurs in over 50% of patients with sepsis, characterized by reversible left ventricular dilatation and impaired systolic or diastolic ventricular dysfunction [1–3], and is strongly associated with a sequential decline in cardiac output and ischemic injury to vital organs [4]. Despite advancements in antibiotic therapies and intensive care technologies over the past few decades, the optimal management of septic cardiomyopathy remains unclear, with most current interventions yielding limited clinical efficacies [5]. Consequently, elucidating the underlying mechanisms of septic cardiomyopathy is crucial for the development of innovative therapeutic strategies to mitigate this lethal condition.

Mitochondria play a crucial role in energy metabolism in cardiomyocytes, and their dysfunction is a key pathological mechanism underlying septic cardiomyopathy [6]. The inflammatory response triggered by sepsis has been shown to disrupt mitochondrial function, thereby severely impairing cardiac metabolism [7]. Impaired mitochondria accumulate damage-associated molecular patterns (DAMPs) [8], which exacerbate inflammation and activate deleterious pathways, including apoptosis, oxidative stress, and calcium overload, ultimately culminating in myocardial dysfunction [9]. Mitophagy is a critical autophagic process that selectively targets and removes damaged or dysfunctional mitochondria via the lysosomal pathway [10]. Dysregulation of mitophagy has been implicated in various cardiac pathologies, including ischemia–reperfusion injury, heart failure, and septic cardiomyopathy. Activation of mitophagy has been shown to alleviate mitochondrial dysfunction by reducing the inflammatory response, oxidative stress, mitochondrial permeability transition pore (mPTP) opening, and mitochondrial apoptosis [11]. Targeting mitophagy may offer a promising therapeutic strategy for improving cardiac outcomes and reducing mortality in patients with septic cardiomyopathy.

Receptor-mediated mitophagy is a crucial regulatory pathway for mitophagy, comprising key components such as BCL2/adenovirus E1B 19 kDa protein-interacting protein 3 (Bnip3) and FUN14 domain containing 1 (FUNDC1) [12]. FUNDC1, a mitochondrial outer membrane protein, interacts with endoplasmic reticulum (ER) proteins and plays a significant role in regulating ER–mitochondrial interactions, including membrane formation and calcium exchange between the ER and mitochondria [13,14]. Notably, FUNDC1-mediated mitophagy has been implicated in early stages of hypoxia during ischemic preconditioning [15]. Our previous studies have demonstrated that FUNDC1-mediated mitophagy is essential for maintaining mitochondrial homeostasis in septic cardiomyopathy [16]. Therefore, further investigation is warranted to explore the potential of modulating this pathway as a novel approach for developing effective therapies.

Fibroblast growth factor 2 (FGF2) plays a crucial role in cellular proliferation, differentiation, and survival and has been implicated in the development of various vascular diseases, including myocardial infarction, atherosclerosis, and pulmonary hypertension [17]. Recent studies have investigated its impact on mitochondrial function, a critical factor in maintaining cellular energy balance and regulating apoptosis. In cardiac cells, FGF2 has been shown to protect subsarcolemmal mitochondria from calcium-induced permeability transition, thereby preventing cell death through a mechanism involving protein kinase C (PKC) and connexin 43 (Cx43) phosphorylation [18]. FGF2 has also been shown to protect mitochondria by increasing resistance to calcium overload-induced mPTP opening, which is a critical event in mitochondrial-mediated apoptosis [19]. However, the potential of FGF2 to alleviate mitochondrial damage remains poorly understood. FGF2 has also been shown to regulate autophagy, which is a process that can influence mitophagy. In the context of cisplatin-induced granulosa cell injury, FGF2 promotes cell survival by enhancing autophagy, which in turn reduces apoptosis [20]. Therefore, this study aimed to investigate the effects of FGF2 on septic cardiomyopathy, with a focus on FUNDC1-mediated mitophagy.

## Results

### FGF2 expression is significantly decreased in cardiomyocyte during septic stress

**Fig. 1.**
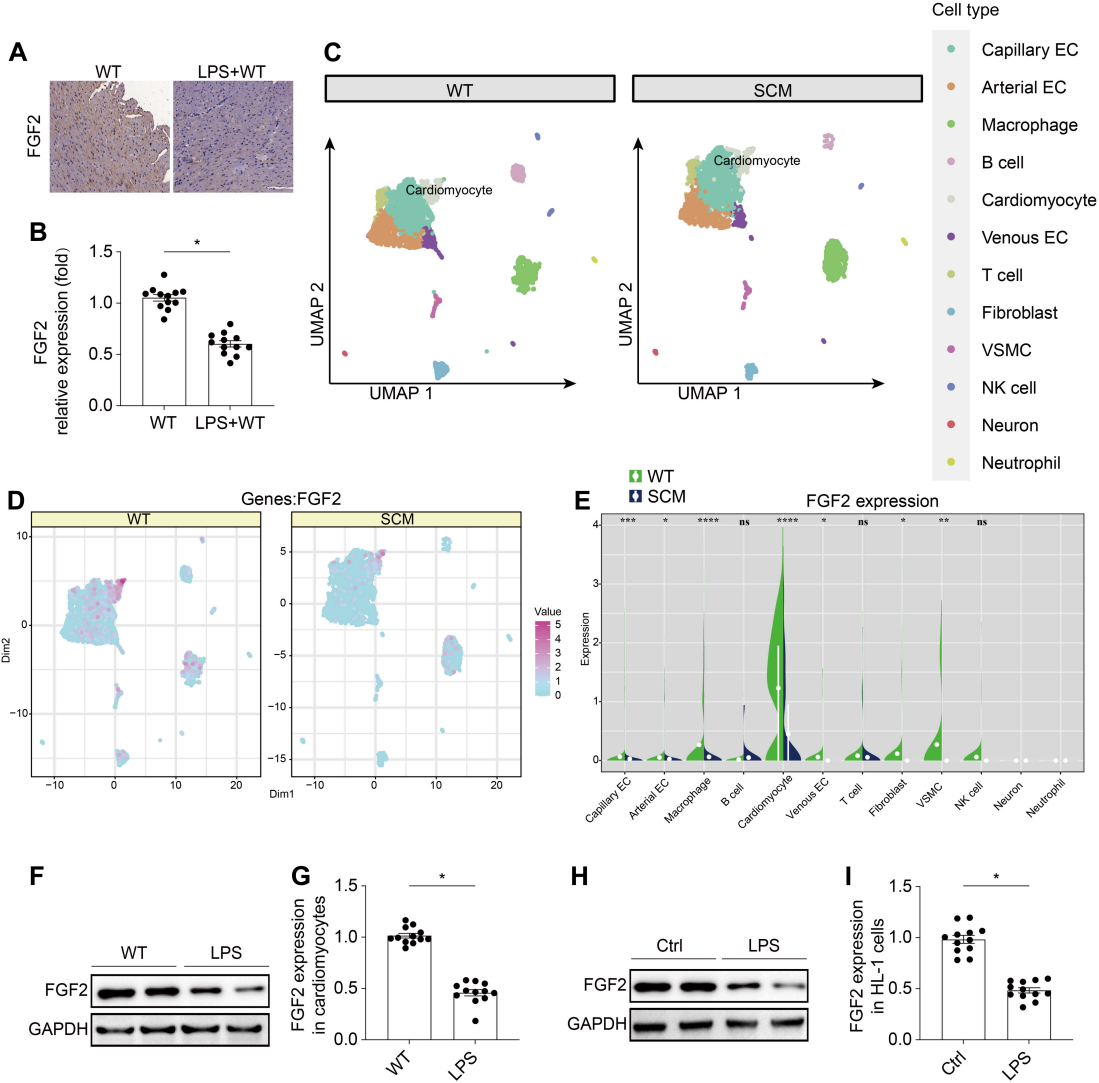
LPS inhibits FGF2 expression in cardiomyocyte. LPS was injected into WT mice to establish the septic cardiomyopathy model. (A and B) Immunohistochemistry of the FGF2 expression decreasing in response to LPS challenge. (C to E) FGF2 expression in different cell types from heart was evaluated using single-cell sequencing analysis. The expression of FGF2 in cardiomyocyte (F and G) and HL-1 (H and I) cells was detected via Western blotting. Data are shown as the means ± SEM (*n* = 12 per group). *P < 0.05.

To investigate the role of FGF2 in septic cardiomyopathy, we established the septic murine model and examined the FGF2 expression in heart tissue via immunohistochemistry. Compared to the sham group, FGF2 expression was significantly decreased following LPS treatment (Fig. 1A and B). To identify the primary cellular source of FGF2 in cardiac tissue, we reanalyzed existing single-cell RNA sequencing data from mouse myocardium, comparing samples with and without septic stress [21]. The results revealed that cardiomyocytes were the primary source of FGF2 expression (Fig. 1C to E and Fig. S1A and B). To further evaluate the impact of septic stress on FGF2 expression in cardiomyocyte, we extracted cardiomyocytes from wild-type (WT) mice and exposed them to lipopolysaccharide (LPS) for a period of 72 h, mimicking the conditions of septic cardiomyopathy in vitro. Western blotting analyses showed that FGF2 expression was significantly reduced in cardiomyocytes under LPS treatment (Fig. 1F and G). The similar results were observed in HL-1 myocardial cells (Fig. 1H and I). These findings suggest that FGF2 may be a key player in the development of septic cardiomyopathy, warranting further investigation into its potential therapeutic applications.

### FGF2 knockout exacerbates cardiac injury in septic cardiomyopathy

**Fig. 2.**
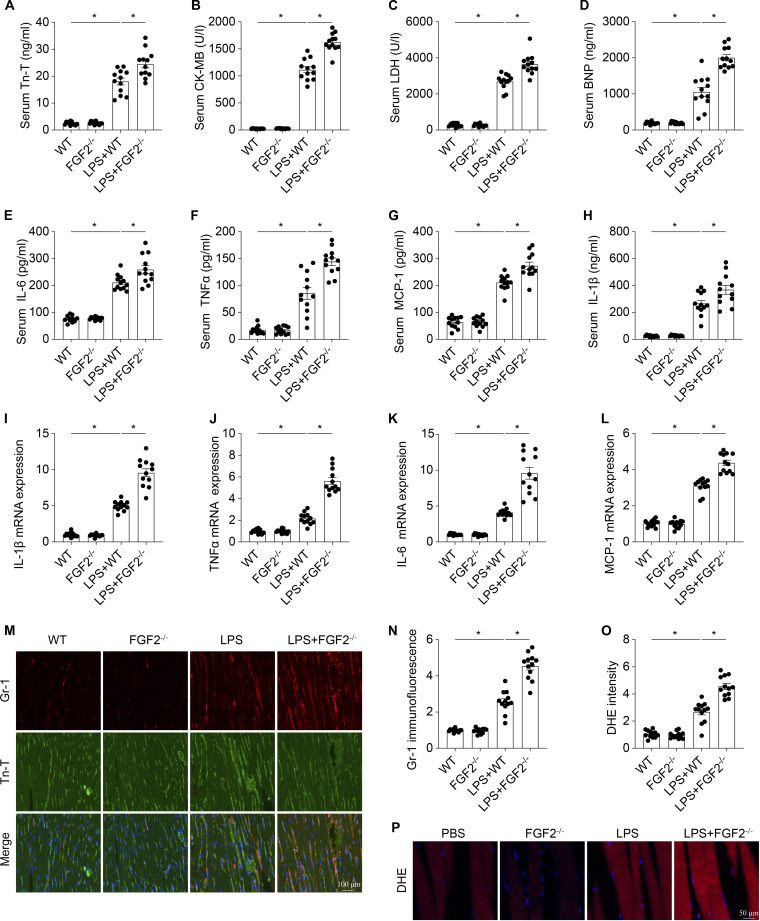
FGF2 deletion aggravates cardiac damage in sepsis. The cardiac injury markers Tn-T, CK-MB, LDH, and BNP (A to D) and the concentrations of inflammatory factors TNFα, IL-6, MCP-1, and IL-β (E to H) in blood were measured via ELISA. (I to L) Following septic cardiomyopathy induced by LPS, the expression levels of mRNAs encoding inflammatory factors, including IL-1β, TNFα, IL-6, and MCP-1, were quantified by qPCR. (M and N) Heart tissues were collected and subsequently subjected to immunofluorescence labeling to examine the aggregation of Gr-1-positive cells in the myocardium. (O and P) Cardiac reactive oxygen species (ROS) content change measured with DHE. Data are shown as the means ± SEM (*n* = 12 per group). *P < 0.05.

To explore whether reduced FGF2 influenced the pathology of septic cardiomyopathy, FGF2 knockout (FGF2^−/−^) mice were used to establish the septic murine model. Cardiac injury markers in the serum, including troponin T (Tn-T), creatine kinase-MB (CK-MB), lactate dehydrogenase (LDH), and B-type natriuretic peptide (BNP), were measured by enzyme-linked immunosorbent assay (ELISA) (Fig. 2A to D). LPS treatment resulted in a significant increase in cardiac injury biomarkers, suggesting enhanced cardiac damage when compared to the sham group. Moreover, FGF2-deficient mice displayed even higher levels of these biomarkers than WT mice, implying an enhanced susceptibility to cardiac injury following LPS treatment. Given that the inflammatory response and oxidative stress are important causes of myocardial injury in septic cardiomyopathy, we then assessed the effect of FGF2 on them. As shown in Fig. 2E to H, LPS treatment significantly elevated the level of proinflammatory factor, including interleukin-6 (IL-6), tumor necrosis factor α (TNFα), MCP-1, and IL-1β, in serum. In vitro experiments using quantitative polymerase chain reaction (qPCR) in cardiomyocytes further confirmed these results (Fig. 2I to L). Compared to the normal cardiomyocytes, LPS treatment significantly up-regulated the mRNA expression of IL-1β, TNFα, IL-6, and MCP-1, where this inflammatory response was further intensified in the absence of FGF2. Immunofluorescence staining was subsequently employed to assess the inflammatory response in the myocardium following LPS treatment. Compared with the LPS group, FGF2 ablation led to a marked increase in the deposition of Gr-1^+^ neutrophils within the myocardium (Fig. 2M and N). Dihydroethidium (DHE) staining was used to estimate the changes in cardiac reactive oxygen species (ROS) content. As shown in Fig. 2O and P, LPS significantly increased the ROS content in cardiac tissue. The level of ROS in the heart of FGF2^−/−^ mice was markedly higher than that in WT mice under septic stress. These results showed that FGF2 gene deletion aggravates myocardial injury in septic cardiomyopathy.

### The absence of FGF2 exacerbates cardiac dysfunction in septic cardiomyopathy

**Fig. 3.**
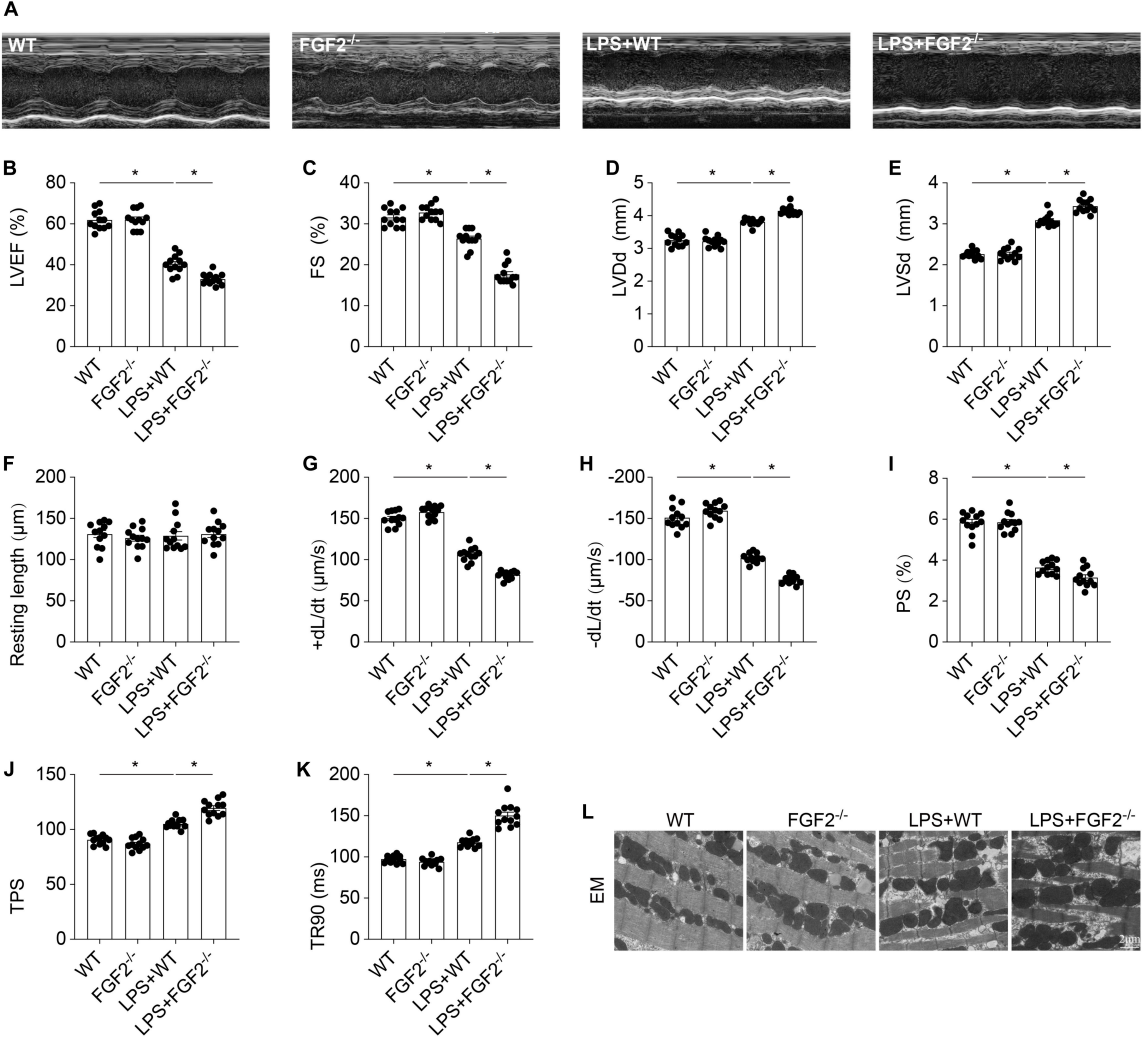
FGF2 deletion promotes cardiac dysfunction in septic cardiomyopathy. (A) Representative M-mode echocardiographic images. (B) Quantification of left ventricular ejection fraction (LVEF). (C) Fractional shortening (FS). (D) Left ventricular end-diastolic diameter (LVDd). (E) Left ventricular end-systolic diameter. (F) Resting cell length. (G) Maximal velocity of shortening (+dL/dt). (H) Maximal velocity of relengthening (−dL/dt). (I) Peak shortening (PS) (normalized to cell length). (J) Time-to-peak shortening (TPS). (K) Time-to-90% relengthening (TR90). (L) Transmission electron microscopy (TEM) was used to observe the ultrastructural changes after LPS treatment in vivo. Data are shown as the means ± SEM (*n* = 12 per group). *P < 0.05.

We then examined the indices associated with cardiac function using echocardiography, including left ventricular ejection fraction (LVEF), fractional shortening (FS), left ventricular end-diastolic diameter (LVDd), and left ventricular systolic diameter (LVSd) (Fig. 3A). As shown in Fig. 3B to E, FGF2^−/−^ mice exhibited significantly reduced cardiac function compared to WT mice following LPS treatment. Then, contractile parameters were analyzed in freshly isolated cardiomyocytes from FGF2^−/−^ and WT mice, and no significant differences in resting length were observed among the groups (Fig. 3F). LPS treatment markedly impaired both contractile and diastolic functions of cardiomyocytes, as evidenced by decreased peak shortening (PS) and ±dL/dt, along with prolonged time to 90% relaxation (TR90) and time to peak shortening (TPS) (Fig. 3G to K). While FGF2 knockout did not alter the tested mechanical parameters under baseline conditions, it exacerbated LPS-induced cardiomyocyte dysfunction. To investigate the structural basis of these functional impairments, electron microscopy was employed, revealing significant ultrastructural changes in the myocardium post-LPS challenge (Fig. 3L). Specifically, myocardial tissues from FGF2^−/−^ mice under septic stress displayed enlarged mitochondria, vacuolization, and disintegration of myofibrils, in contrast to those from WT mice. These findings indicate that FGF2 deletion exacerbates cardiac dysfunction in septic cardiomyopathy.

### FGF2 deficiency potentiates cardiomyocyte mitochondrial dysfunction in septic cardiomyopathy

**Fig. 4.**
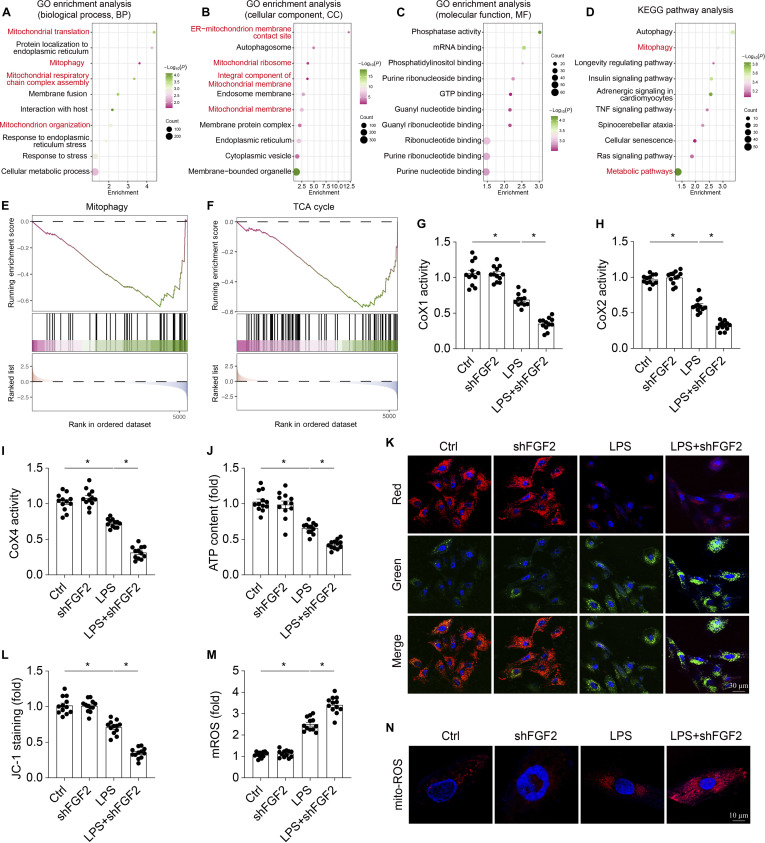
FGF2 ablation potentiates mitochondrial dysfunction. Comparative analysis of cardiac tissue between FGF2^−/−^ mice and WT mice after LPS administration (*n* = 4 per group) revealed 447 differentially expressed proteins (|log_2_FC| > 0.5), with 73 up-regulated and 374 down-regulated proteins. (A to C) Gene Ontology Enrichment Analysis Investigation of the proteins that are expressed at varying levels in heart tissue. (D) KEGG analysis. (E and F) GSEA analysis of differentially expressed genes following FGF2 deletion. (G to I) The activities of the mitochondrial respiratory complexes were quantified using ELISAs. (J) ATP generation. The mitochondrial membrane potential (K and L) and mROS (M and N) were analyzed with an immunofluorescence assay. Data are shown as the means ± SEM (*n* = 12 per group). *P < 0.05.

To elucidate the mechanism by which FGF2 deficiency exacerbates septic cardiac dysfunction, unbiased proteomics analysis from heart tissue of LPS-treated FGF2^−/−^ mice and WT mice was performed (Fig. 4A to F). Functional enrichment analysis revealed that the differentially expressed proteins were enriched in biological processes related to mitochondrial function and metabolism, including mitochondrial translation, mitophagy, and respiratory chain complex assembly (Fig. 4A). Cellular component analysis further indicated that these proteins were predominantly localized to ER–mitochondrial membrane contact site, autophagy, mitochondrial ribosome, integral component of mitochondrial membrane, and mitochondrial protein complex (Fig. 4B). Moreover, pathway analysis using the Kyoto Encyclopedia of Genes and Genomes (KEGG) database revealed that FGF2 deficiency in heart tissue following LPS treatment resulted in significant alterations in mitophagy and metabolic pathways, underscoring a critical role for FGF2 in regulating mitochondrial quality control and energy metabolism (Fig. 4D). Gene Set Enrichment Analysis (GSEA) demonstrated that LPS-treated FGF2^−/−^ mice exhibited reduced mitophagy and tricarboxylic acid cycle (TAC) compared with LPS-treated WT mice (Fig. 4E and F). In summary, the absence of FGF2 disrupts the regulation of proteins essential for maintaining mitochondrial integrity and function in cardiomyocytes under septic stress, highlighting a crucial role for FGF2 in preserving mitochondrial homeostasis in septic cardiomyopathy.

We subsequently conducted a series of validation experiments to further elucidate the impact of FGF2 depletion on mitochondrial homeostasis. To assess mitochondrial function, we measured mitochondrial complex activity (Fig. 4G to I), adenosine triphosphate (ATP) production (Fig. 4J), mitochondrial membrane potential (Fig. 4K and L), and mitochondrial ROS (mROS) (Fig. 4M and N). The results demonstrated significant reductions in these parameters in FGF2 knockdown HL-1 cells following LPS treatment compared to the control group. These findings suggest that the loss of FGF2 exacerbates cardiac injury by aggravating mitochondrial dysfunction in septic cardiomyopathy.

### Genetic ablation of FGF2 potentiates cardiomyocyte death in response to septic stress

**Fig. 5.**
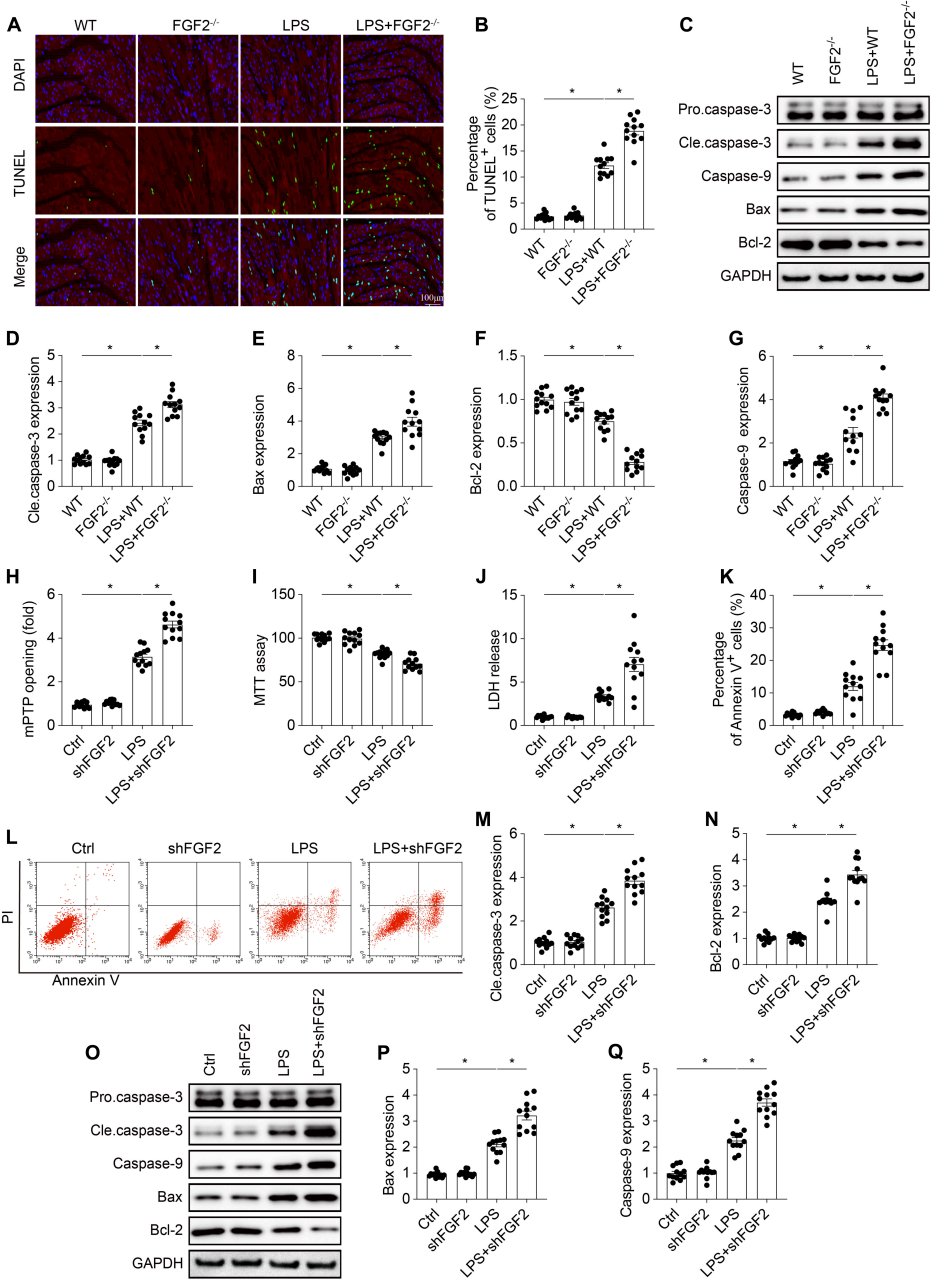
FGF2 deficiency promotes cardiomyocyte apoptosis under septic stress in vivo and in vitro. (A) Representative images of TUNEL staining (green) for apoptotic cells and DAPI (blue) for nuclei in cardiac tissue sections. Scale bar, 100 μm. (B) Quantification of the percentage of TUNEL-positive cells. (C) Representative Western blots showing protein levels of pro-caspase-3, cleaved caspase-3, caspase-9, Bax, and Bcl-2 in cardiac tissue. (D to G) Quantification of the relative protein expression of cleaved caspase-3, Bax, Bcl-2, and caspase-9. (H) mPTP opening was determined by calcein retention. (I) Cell viability assessed by MTT assay. (J) LDH release. (K and L) Representative flow cytometry plots showing Annexin V/PI staining for apoptotic cells. (M to Q) Representative Western blots showing protein levels of pro-caspase-3, cleaved caspase-3, caspase-9, Bax, and Bcl-2 in cell lysates. Data are shown as the means ± SEM (*n* = 12 per group). *P < 0.05.

Mitochondrial damage is a well-established trigger for apoptosis, and therefore, we investigated the impact of FGF2 on cardiomyocyte apoptosis under septic stress. To assess cellular apoptosis in cardiac tissue, we performed terminal deoxynucleotidyl transferase-mediated deoxyuridine triphosphate nick end labeling (TUNEL) assays on cardiac tissue from LPS-treated mice. The results showed a significant increase in TUNEL-positive cells in the hearts of LPS-treated mice, which was further exacerbated in FGF2 knockout mice (Fig. 5A and B). Moreover, FGF2 knockout was associated with a significant up-regulation of proapoptotic proteins, including cleaved caspase-3, caspase-9, and Bax, and a concomitant down-regulation of the anti-apoptotic protein Bcl-2, compared to WT mice subjected to LPS challenge (Fig. 5C to G). To provide more detailed data, in vitro experiments were also accomplished. As shown in Fig. 5H, LPS significantly increased mPTP opening time in HL-1 cells, an effect that was further exacerbated by FGF2 knockdown. The cellular apoptosis index, as assessed by methylthiazolyldiphenyl-tetrazolium bromide (MTT) assay (Fig. 5I), LDH release assay (Fig. 5J), and Annexin V/propidium iodide (PI) staining (Fig. 5K and L), was significantly elevated in HL-1 cells, with a markedly higher increase in the shFGF2 transfection group following LPS stimulation. Furthermore, the absence of FGF2 resulted in higher expression levels of proapoptotic proteins in HL-1 cells under LPS treatment (Fig. 5M to Q). Collectively, these results imply that FGF2 deletion promotes cardiomyocyte apoptosis in septic cardiomyopathy.

### FGF2 supplementation attenuates cardiac injury and dysfunction against septic stress

**Fig. 6.**
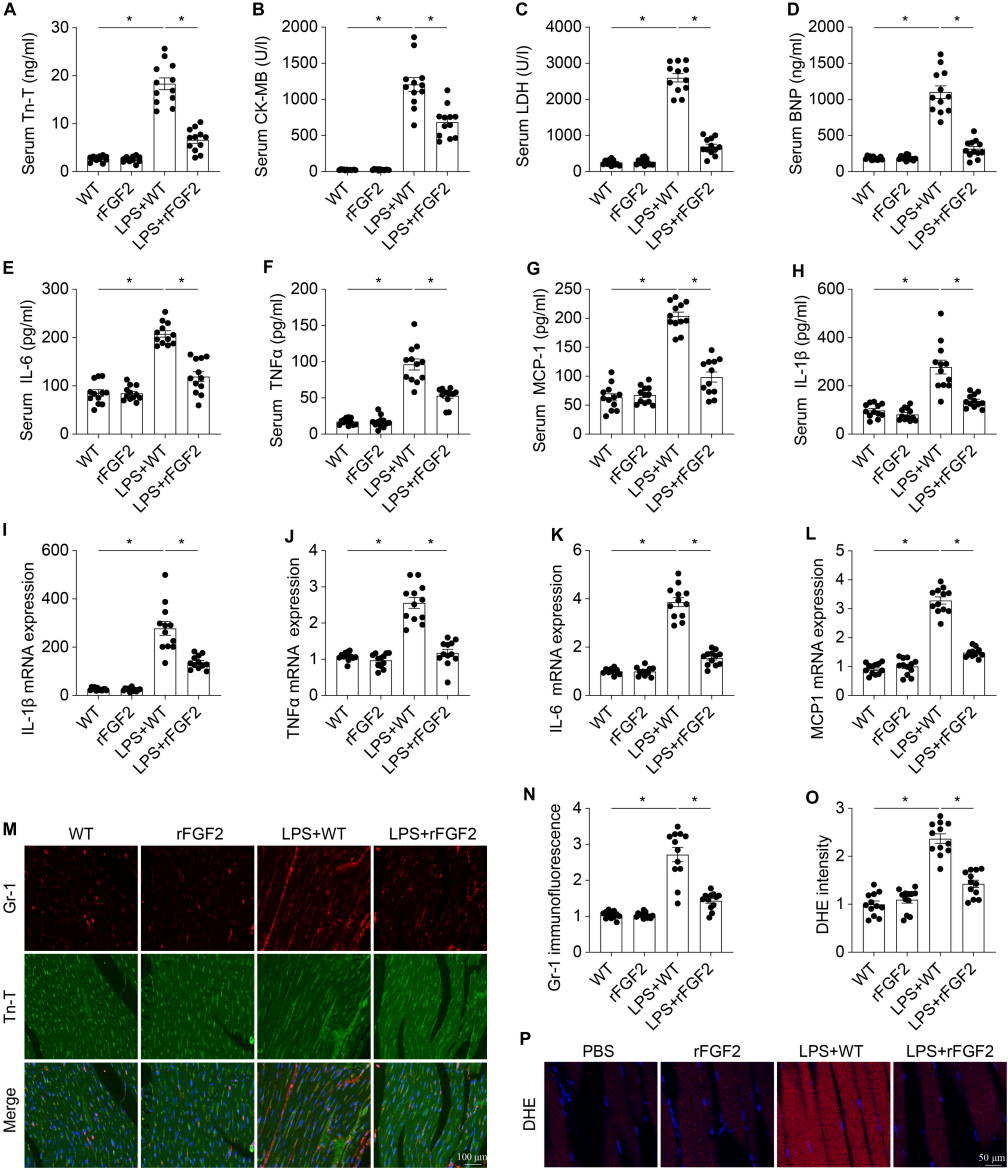
rFGF2 attenuates cardiac damage in sepsis-induced cardiomyopathy in vivo. The cardiac injury markers Tn-T, CK-MB, LDH, and BNP (A to D) and the concentrations of inflammatory factors TNFα, IL-6, and MCP-1 (E to H) in blood were measured via ELISA. (I to L) After LPS-mediated septic cardiomyopathy, the level of mRNAs encoding inflammatory factors, such as IL-1β, TNFα, IL-6, and MCP-1 was measured via qPCR. (M and N) Heart tissues were collected, and then immunofluorescence staining was performed to analyze the accumulation of Gr-1-positive cells in the myocardium. (O and P) Cardiac ROS content change measured with DHE. Data are shown as the means ± SEM (*n* = 12 per group). *P < 0.05.

To strengthen and confirm the protective effect of FGF2 in sepsis-induced cardiac injury, mice were administered 25 μg of recombinant murine FGF2 protein (rFGF2). Treatment with rFGF2 prevented the up-regulation of cardiac injury markers (Fig. 6A to D), mitigated the inflammatory response (Fig. 6E to N), and reduced ROS generation under septic stress (Fig. 6O and P). Echocardiographic analysis demonstrated that rFGF2 significantly improved cardiac function in comparison to WT mice following LPS treatment (Fig. S2A to D). Furthermore, rFGF2 markedly alleviated LPS-induced contractile and diastolic dysfunctions in cardiomyocytes (Fig. S2E to J). Structural analysis revealed that rFGF2 ameliorated myocardial ultrastructural disruptions induced by LPS, as evidenced by reduced myocardial degradation and preservation of Z-line integrity (Fig. S2K). In addition, the impact of FGF2 supplementation on cardiomyocyte apoptosis was assessed. rFGF2 treatment significantly decreased the number of TUNEL-positive cells (Fig. S3A and B) and the expression of proapoptotic proteins (cleaved caspase-3, caspase-9, Bax, and Bcl-2) in cardiac tissue compared to the WT group (Fig. S3C to G).

These protective effects were recapitulated in vitro using HL-1 cardiomyocytes. To determine the optimal rFGF2 concentration, cells were pretreated with varying concentrations of rFGF2 (0, 12.5, 25, 50, and 100 ng/ml) for 1 h prior to LPS challenge for 72 h. Cell viability assessed by Cell Counting Kit-8 (CCK-8) assay revealed that 25 ng/ml rFGF2 provided maximal cytoprotection against LPS-induced injury (Fig. S3H). This concentration was therefore used in all subsequent experiments. rFGF2 supplementation significantly reduced cellular apoptosis, as evidenced by LDH release (Fig. S3I) and Annexin V/PI flow cytometry analysis (Fig. S3J and K). The anti-apoptotic effect of rFGF2 was further confirmed by the down-regulation of proapoptotic proteins (caspase-3, caspase-9, and Bax/Bcl-2) in HL-1 cells under LPS challenge (Fig. S3L to P). Collectively, these findings suggest that FGF2 supplementation confers protection against cardiac injury and preserves cardiac function during sepsis by mitigating inflammation, oxidative stress, and apoptosis.

### FGF2 potentiates mitophagy under septic stress

**Fig. 7.**
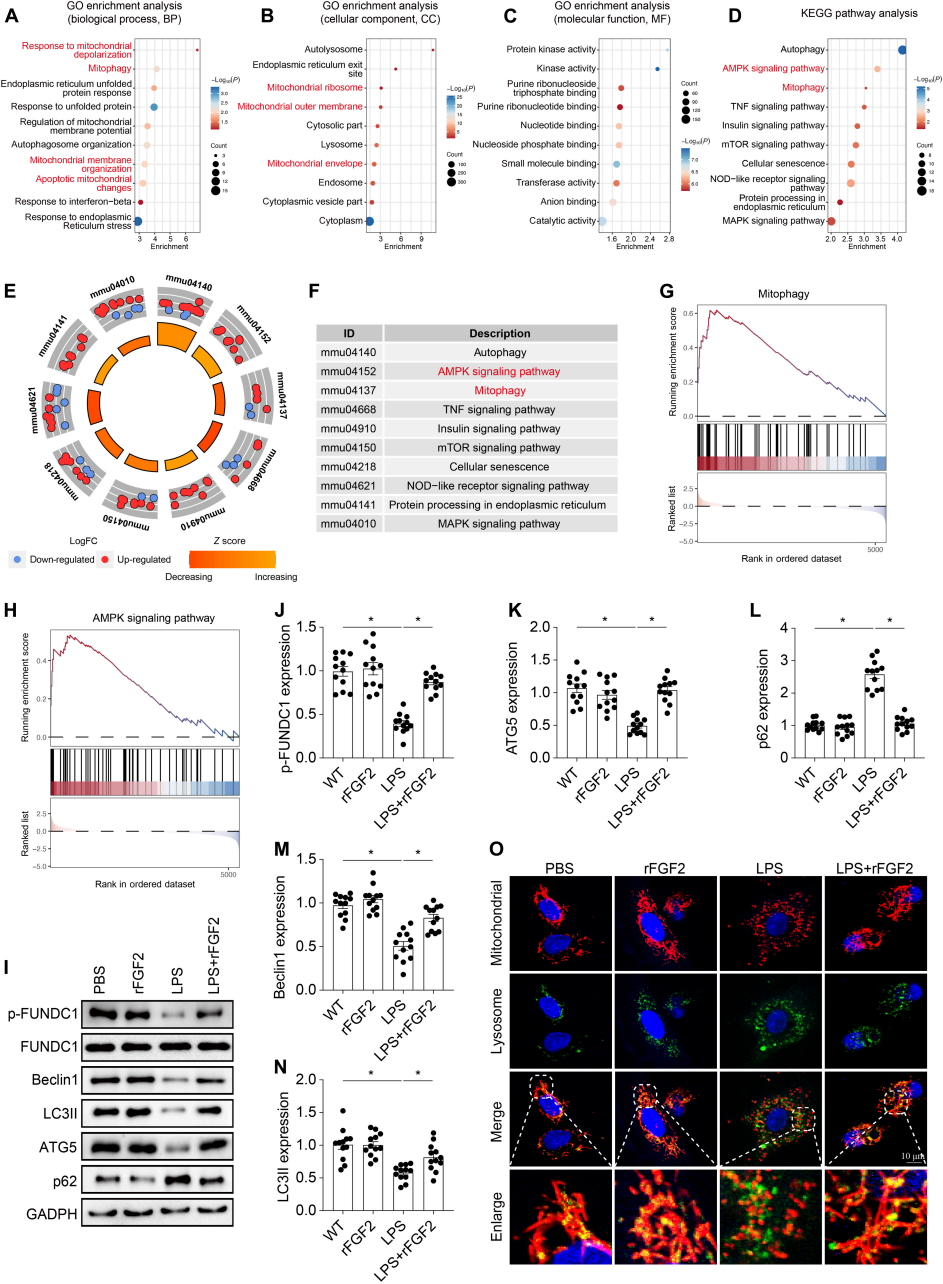
rFGF2 ameliorates LPS-induced mitochondrial damage by activating mitophagy. Comparative analysis of cardiac tissue between rFGF2-treated and WT mice after LPS administration (*n* = 4 per group) revealed 444 differentially expressed proteins (|log_2_FC| > 0.5), with 328 up-regulated and 116 down-regulated proteins. (A to C) Gene Ontology Enrichment Analysis of differentially expressed proteins in cardiac tissue. (D to F) KEGG analysis. (G and H) GSEA visual analysis of mitophagy-related pathways and AMPK pathway in cardiac tissue. (I to N) The expression of p-FUNDC1, Beclin1, LC3II, ATG5, and p62 was measured via Western blotting. (O) Co-immunofluorescence of mitochondria and lysosomes in HL-1 cells. Data are shown as the means ± SEM (*n* = 12 per group). *P < 0.05.

To elucidate the molecular mechanisms underlying FGF2’s protective effects against septic cardiomyopathy, we employed a proteomics approach, analyzing heart tissue from mice treated with rFGF2 and phosphate-buffered saline (PBS) under LPS challenge, to identify differentially regulated proteins and signaling pathways involved in this process (Fig. 7A to D). Functional enrichment analysis revealed that the differentially expressed proteins were enriched in biological processes related to response to mitochondrial depolarization, mitophagy, regulation of mitochondrial membrane potential, mitochondrial membrane organization, and apoptotic mitochondrial changes (Fig. 7A). Cellular component analysis showed that these proteins were primarily localized to mitochondrial ribosome, mitochondrial outer membrane, and mitochondrial envelope (Fig. 7B). Moreover, KEGG analysis revealed that mitophagy and adenosine monophosphate-activated protein kinase (AMPK) signaling pathway were among the top enriched pathways (Fig. 7D to F), and GSEA further confirmed that mitophagy and AMPK signaling were significantly induced by rFGF2 treatment (Fig. 7G and H).

Our previous study highlighted the significance of the FUNDC1-mediated mitophagy pathway in protecting heart function and maintaining mitochondrial integrity [22]. Considering the findings from the enrichment analysis, we investigated whether FGF2 modulates mitochondrial bioenergetics by activating FUNDC1-mediated mitophagy. Firstly, the expression levels of p-FUNDC1, LC3II, Atg5, Beclin1, and p62 in cardiac tissue from WT and FGF2 knockout mice following LPS challenge were measured by Western blotting. Following LPS administration, WT mice showed a noticeable decrease in markers associated with mitophagy activation as indicated by decreased expression of p-FUNDC1, LC3II, Atg5, and Beclin1 while increasing the expression of p62 (Fig. S4A to F). However, this suppression was significantly exacerbated in FGF2^−/−^ mice treated with LPS. Experimental validation in HL-1 cells provided further confirmation that FGF2 is critical for maintaining robust mitophagy under LPS stress. As shown in Fig. 7J to N, LPS treatment significantly decreased the expression of p-FUNDC1, LC3II, Atg5, and Beclin1 while increasing the expression of p62. This effect was reversed by rFGF2 treatment. Additionally, co-immunofluorescence was performed to determine the relationship between mitochondria and lysosomes in HL-1 cells exposed to LPS in vitro. We found that rFGF2 facilitated the colocalization of mitochondria and lysosomes following LPS treatment (Fig. 7O). These results suggest that FGF2 reactivates FUNDC1-dependent mitophagy in septic cardiomyopathy, thereby potentially modulating mitochondrial bioenergetics and protecting cardiac function.

### FGF2 inhibits septic stress-induced mitochondrial dysfunction via activating mitophagy

**Fig. 8.**
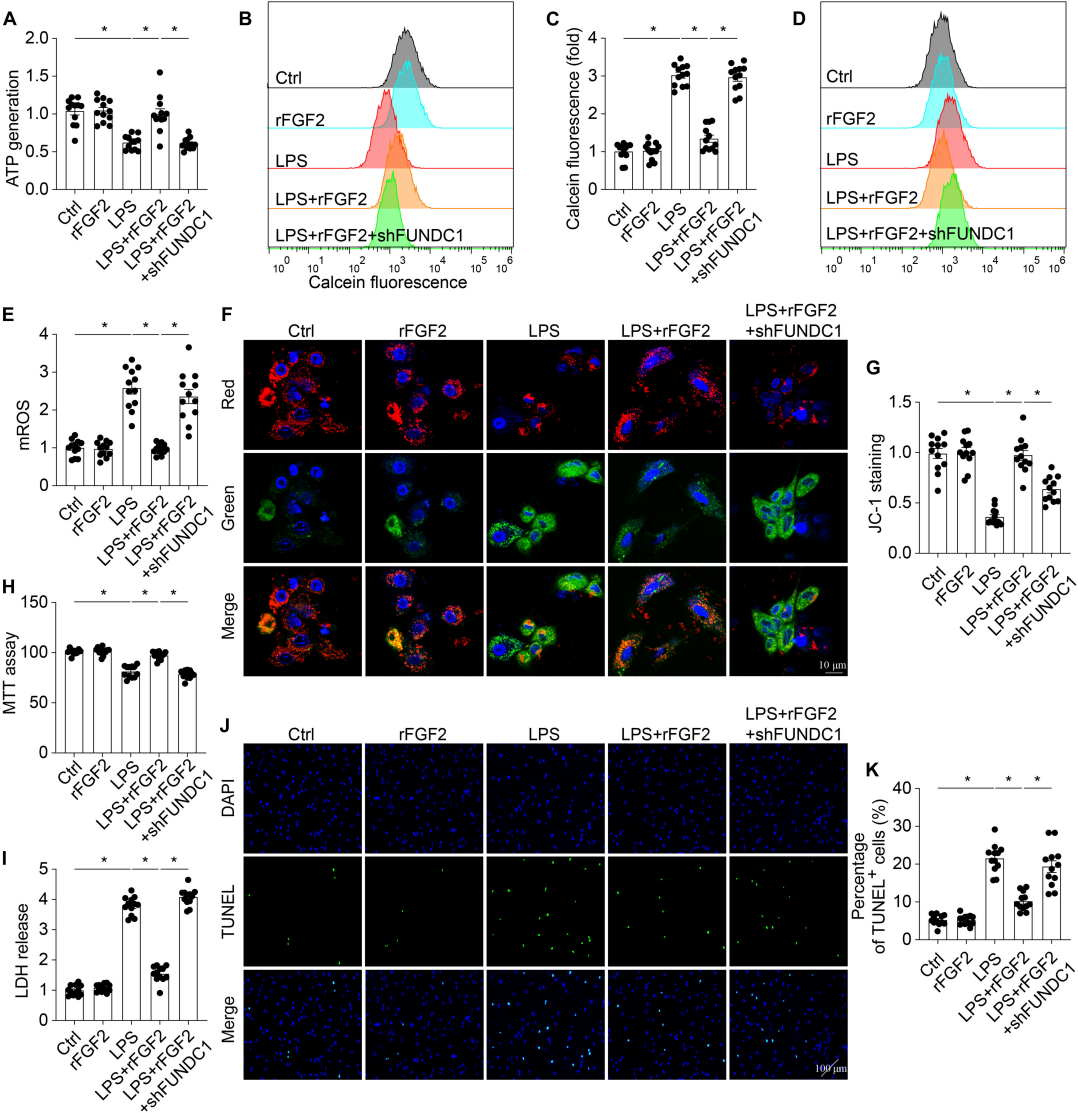
rFGF2 activated FUNDC1-required mitophagy, which was inhibited in response to LPS. (A) ATP generation. (B and C) HL-1 cells were incubated with the reagents in the MitoProbe Transition Pore Assay Kit and analyzed by flow cytometry. (D and E) mROS were analyzed by flow cytometry. (F and G) The mitochondrial membrane potential was analyzed with an immunofluorescence assay. Cellular viability was measured via MTT assay (H), LDH release (I), and TUNEL assay (J and K). Data are shown as the means ± SEM (*n* = 12 per group). *P < 0.05.

To investigate whether FUNDC1-mediated mitophagy acts as a compensatory mechanism during FGF2 treatment, we used short hairpin RNA (shRNA) to inhibit FUNDC1-mediated mitophagy in FGF2-treated HL-1 cells. rFGF2 treatment significantly alleviated mitochondrial injury, evidenced by increased ATP production (Fig. 8A), decreased mPTP opening time (Fig. 8B and C), reduced mROS generation (Fig. 8D and E), and enhanced mitochondrial membrane potential (Fig. 8F and G). Notably, these protective effects on mitochondrial function were abolished by FUNDC1 inhibition in vitro. Moreover, FUNDC1 knockdown negated the cardioprotective effects of rFGF2, as demonstrated by decreased cellular viability (Fig. 8H), increased LDH release (Fig. 8I), and a higher number of TUNEL-positive cells (Fig. 8J and K) in rFGF2-treated HL-1 cells under LPS challenge. These findings suggest that FUNDC1-mediated mitophagy is a crucial adaptive mechanism through which FGF2 exerts its protective effects against septic cardiomyopathy.

### FGF2 activates FUNDC1 via the AMPK pathway

**Fig. 9.**
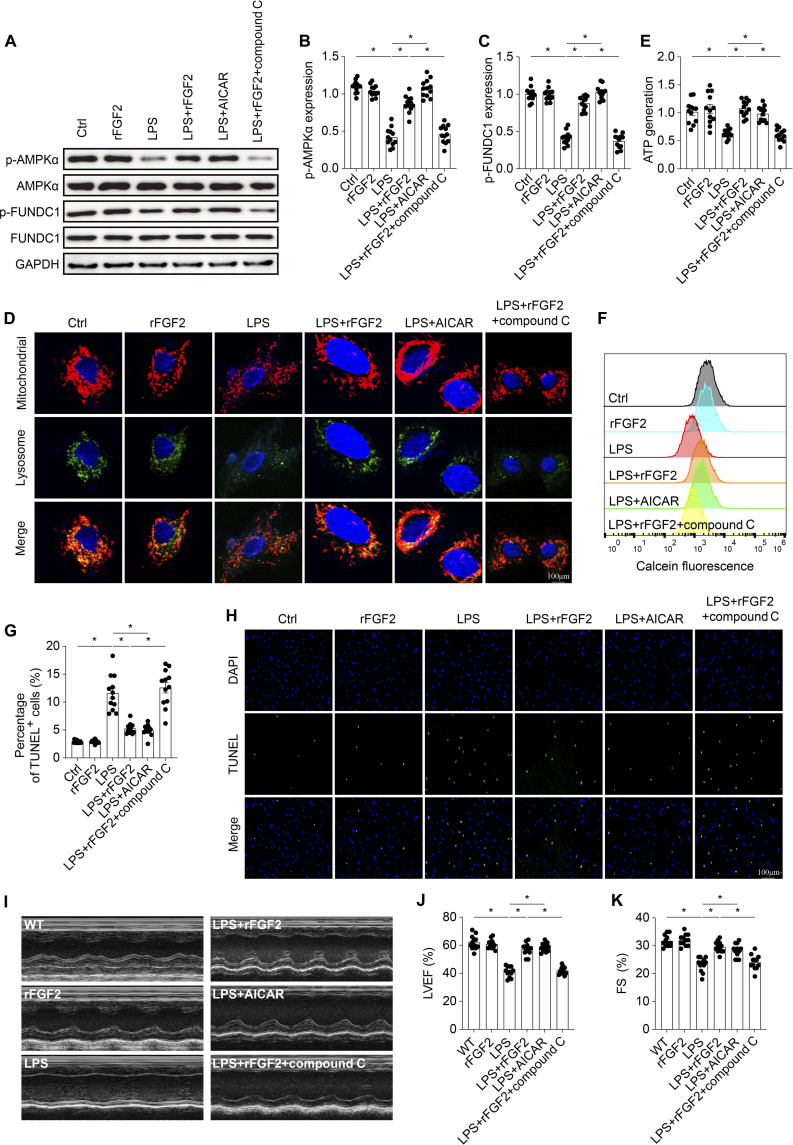
FGF2 supplementation promotes FUNDC1-mediated mitophagy by activating AMPK pathway. (A to C) Western blots were used to analyze the expression change of p-AMPK and p-FUNDC1. (D) Co-immunofluorescence of mitochondria and lysosomes in HL-1 cells. (E) ATP generation. (F) mPTP opening time. (G and H) The cellular apoptosis was measured via TUNEL assay. (I to K) Representative M-mode echocardiographic images and quantification of LVEF and FS in mice from different treatment groups. Data are shown as the means ± SEM (*n* = 12 per group). *P < 0.05.

Our final investigation focused on how FGF2 promotes FUNDC1-dependent mitophagy in cardiomyocytes. KEGG analysis indicated that the AMPK signaling pathway could be a potential downstream effector of FGF2 (Fig. 7H). AMPK is a serine/threonine protein kinase complex known to increase FUNDC1 phosphorylation. LPS challenge caused a reduction in p-AMPKα in cardiac tissue, indicating a suppression of AMPK activity (Fig. S4G and H). This reduction in p-AMPKα levels was significantly more severe in LPS-treated FGF2^−/−^ mice compared to LPS-treated WT mice. We also conducted verification experiments in HL-1 cells treated with LPS. Our results showed that LPS inhibited p-AMPKα expression, which was restored by rFGF2 treatment (Fig. 9A to C). To validate these findings, we used AICAR, a known AMPK pathway activator, as a positive control, and compound C, an AMPK pathway inhibitor, as a negative control. AICAR treatment in the LPS group promoted AMPK phosphorylation, increased p-FUNDC1 expression, colocalization of mitochondria and lysosomes (Fig. 9D), and ATP generation (Fig. 9E), and reduced mPTP opening (Fig. 9F). Additionally, AICAR treatment significantly reduced the number of TUNEL-positive cells in HL-1 cells under LPS challenge (Fig. 9G and H), mirroring the effects observed with rFGF2 treatment. However, compound C negated the cardioprotective effects of rFGF2 in HL-1 cells in response to LPS. Finally, we assessed cardiac function by echocardiography (Fig. 9I to K). LPS administration induced a significant reduction in LVEF and FS, indicative of impaired systolic function. Cotreatment with AICAR significantly ameliorated these functional deficits, restoring LVEF and FS to near-baseline levels comparable to those observed with rFGF2 treatment. Critically, the cardioprotective effects of rFGF2 on both LVEF and FS were largely abolished by co-administration of compound C. These results confirm that FGF2 regulates FUNDC1-related mitophagy via the AMPK pathway.

## Discussion

Septic cardiomyopathy and associated cardiac dysfunction are severe complications of sepsis, characterized by high morbidity and mortality rates [23]. Despite the clinical significance of this condition, the underlying pathophysiology of septic cardiomyopathy remains poorly understood, and therapeutic strategies are lacking. Recent studies have suggested that FGF2 exerts a protective role in cardiac injury. This study employed a combination of loss- and gain-of-function experiments to comprehensively examine the role of FGF2 in septic cardiomyopathy. Our findings indicate that septic stress leads to a significant decrease in FGF2 levels in cardiomyocyte. Notably, the absence of FGF2 was found to exacerbates cardiac dysfunction by inducing mitochondrial damage and promoting cardiomyocyte apoptosis. In contrast, supplementation with FGF2 was shown to mitigate cardiac injury and mitochondrial dysfunction under septic conditions. Furthermore, our research suggests that FGF2 may activate FUNDC1-mediated mitophagy via the AMPK pathway, thereby reducing mitochondrial damage and subsequent apoptosis in response to LPS exposure. To our knowledge, this study provides the first evidence of the mechanism by which FGF2 regulates FUNDC1-mediated mitophagy through the AMPK pathway. This mechanistic link between FGF2 and mitophagy represents a novel finding in the context of sepsis.

FGF2 is a heparin-binding protein that exists in 2 distinct forms: high molecular weight FGF2 (~22 to 24 kDa) and low molecular weight FGF2 (~18 kDa) [24,25]. Different isoforms of FGF2 have distinct effects on mitochondrial function. Low molecular weight FGF2 is primarily cytoplasmic/secreted and signals via the FGF receptor (FGFR) at the cell surface, whereas high molecular weight FGF2 contains additional N-terminal nuclear localization sequences and often acts through intracrine/nuclear mechanisms. The low molecular weight FGF2 isoform has been shown to protect mitochondria by increasing resistance to calcium overload-induced mPTP opening, which is a critical event in mitochondrial-mediated apoptosis [19]. Conversely, high molecular weight FGF2 isoforms can induce mitochondrial-driven apoptosis by interacting with mitochondrial proteins such as C1QBP, highlighting the dual role of FGF2 in mitochondrial regulation [26]. Our study focused primarily on the effects of low molecular weight FGF2 isoforms, but high molecular weight FGF2 isoforms may exhibit differential or even opposing effects on cardiac protection. Whether these isoforms modulate FUNDC1-dependent mitophagy through distinct mechanisms remains an important question for future investigation.

The low molecular weight FGF2 has been shown to possess cardioprotective properties both in vitro and in vivo. Upon external administration, low molecular weight FGF2 interacts with the FGFR1 receptor on the cell membrane, triggering a signaling cascade that prevents the opening of mPTP and the release of cytochrome c during calcium overload [18]. Studies have demonstrated that increased low molecular weight FGF2 expression in cardiac tissue mitigates post-ischemic systolic dysfunction and reduces the severity of myocardial infarction in mice [27]. Analysis of single-cell sequencing data revealed that FGF2 is predominantly produced by cardiomyocytes and may play a crucial role in regulating cardiac function. In this study, we identified FGF2 as a potential therapeutic target for septic cardiomyopathy. Our findings indicate that FGF2 significantly enhances cardiac function and reduces serum levels of myocardial injury markers, including Tn-T, CK-MB, LDH, and BNP. Furthermore, FGF2 was found to mitigate the inflammatory response in LPS-induced septic cardiomyopathy model. Notably, FGF2 markedly decreased cardiomyocyte apoptosis under LPS challenge both in vivo and in vitro, as evidenced by TUNEL staining and reduced expression of proapoptotic proteins. Additionally, FGF2 protected mitochondrial function in cardiomyocytes from damage caused by oxidative stress, disruptions in energy metabolism, and cytotoxic metabolites associated with septic cardiomyopathy. These findings suggest that FGF2 exerts direct protective effects on cardiomyocytes by preserving mitochondrial integrity and restricting inflammation, highlighting its potential as a therapeutic agent for the treatment of septic cardiomyopathy. While our findings highlight the cardioprotective role of FGF2, its direct therapeutic application faces key limitations. FGF2 has a short half-life and may induce off-target angiogenic or proliferative effects due to its pleiotropic nature [28,29]. Frequent systemic administration may raise feasibility and safety concerns. Emerging strategies, including nanoparticle-based delivery and controlled-release systems, have shown promise in enhancing cardiac specificity and stability [30,31]. Importantly, our study emphasizes FGF2-regulated signaling as a therapeutic axis. Future development of isoform-selective agonists or small-molecule modulators may overcome translational barriers and enable clinical application.

Our previous research underscored the crucial role of mitophagy in sepsis-induced cardiomyopathy, which is characterized by a reduction in mitochondrial membrane potential, an increase in mROS, and enhanced cardiomyocyte apoptosis [32]. Although it is well established that mitophagy is regulated by various receptors, including FUNDC1, Bnip3, and Parkin [22,33,34], the upstream mechanisms underlying mitophagy inactivation in septic cardiomyopathy remained poorly understood. FUNDC1 has been identified as a key mitophagy receptor in mammalian cells [35]. Under hypoxic conditions, reduced phosphorylation of FUNDC1 is strongly correlated with the initiation of mitophagy [35]. This, in turn, leads to diminished myocardial contractility and elevated injury markers, ultimately resulting in cardiac dysfunction [16]. Our research demonstrates that the protective effect of FGF2 on mitophagy in septic cardiomyopathy is mediated by FUNDC1, providing novel insight into the underlying mechanisms of sepsis-induced cardiac dysfunction. This breakthrough finding has significant implications for the development of potential therapeutic interventions targeting the FGF2–FUNDC1–mitophagy axis, which may ultimately lead to improved treatment strategies for septic cardiomyopathy.

Previous studies have implicated the AMPK pathway in the regulation of FUNDC1-mediated mitophagy in post-myocardial infarction models. However, the specific role of FGF2 in septic cardiomyopathy, particularly its interaction with FUNDC1-associated mitophagy, has remained unclear until recently. AMPK is a well-established key signaling pathway that plays a crucial role in mitigating oxidative stress and inflammation, thereby protecting against ischemia–reperfusion injury. FGF2, a known endogenous cardioprotective factor, has been shown to activate various physiological processes through the AMPK pathway in multiple tissues and disease contexts. Our study has now revealed that FGF2 regulates FUNDC1-related mitophagy via the AMPK pathway in the context of septic cardiomyopathy, providing a novel insight into the pathophysiology of sepsis-induced cardiac dysfunction. This finding builds upon and expands previous studies, establishing a new connection between FGF2, AMPK signaling, and FUNDC1-mediated mitophagy in the development of septic cardiomyopathy. Indeed, FGF2 is well established to activate canonical prosurvival cascades, including mitogen-activated protein kinase (MAPK)/extracellular signal–regulated kinase (ERK), phosphatidylinositol 3-kinase (PI3K)–Akt, and PKC signaling pathways, which may act synergistically with mitophagy to preserve mitochondrial integrity and cellular function [36,37]. Future studies dissecting the interplay between FUNDC1-mediated mitophagy and alternative FGF2 downstream cascades will be essential to provide a more comprehensive mechanistic framework.

Several limitations of our study merit acknowledgment. First, the LPS-induced sepsis model, while offering experimental controllability and reproducibility, represents only one aspect of the complex septic syndrome. Clinical sepsis involves diverse pathogens, varying immune responses, and dynamic pathophysiological changes that may not be fully recapitulated by endotoxin administration alone [38,39]. Future validation using polymicrobial models such as cecal ligation and puncture will be necessary to confirm the broader applicability of our findings across different septic contexts. Second, while our primary cardiomyocyte experiments support the key findings observed in HL-1 cells, inherent differences between immortalized cell lines and native cardiac tissue may influence cellular responses to septic stress. Additionally, our global FGF2 knockout approach cannot exclude potential developmental compensations or indirect effects through noncardiac tissues. Future studies employing inducible, cardiomyocyte-specific FGF2 deletion models will be essential to definitively establish the cell-autonomous role of FGF2 in septic cardiomyopathy and to minimize confounding factors associated with constitutive gene deletion.

In conclusion, our study reveals that FGF2 exerts a protective effect in septic cardiomyopathy by regulating FUNDC1-dependent mitophagy, identifying FGF2 as a key upstream regulatory factor of FUNDC1. These findings have important implications for the development of novel therapeutic strategies for the treatment of septic cardiomyopathy, highlighting the potential of targeting the FGF2–FUNDC1 axis as a promising approach for mitigating cardiac dysfunction in sepsis.

## Materials and Methods

### Construction of animal models

FGF2^−/−^ mice (global knockout, 010698, Jackson Laboratory, USA) were bought from Jackson Laboratory as previously described [15] and were kept under normal conditions (temperature of 21 to 25 °C, relative humidity: 45% to 55%, 12-h light/dark period) and unrestricted access to water and food. All animal experiments were performed in accordance with ethical guidelines and approved by the Chinese PLA General Hospital’s Committee (S2023-359-03). To induce septic cardiomyopathy, 6- to 8-week-old mice received an intraperitoneal injection of LPS (*Escherichia coli* 0111: B4, #2630, Sigma-Aldrich, USA) [32]. After 72 h, the animals were humanely euthanized with pentobarbital, and their heart tissues were harvested and preserved for further analysis. In rescue experiments, mice received intravenous injections of 25 μg of recombinant murine FGF2 protein (rFGF2, PeproTech, catalog no. 450-33) 12 h before and 1 and 2 d after LPS administration. The AMPK agonist AICAR (500 mg/kg) and inhibitor compound C (20 mg/kg) were administered intraperitoneally [40,41].

### Cell incubation, induction, and transfection

The HL-1 myocardial cell line was bought from Sigma-Aldrich (catalog no. SCC065) and then incubated at 37 °C and 5% CO_2_ with Dulbecco’s modified Eagle’s medium/F-12 nutrient mixture (DMEM/F-12; Invitrogen, Carlsbad, CA, USA) cultivation system [42]. The isolation and culture of mouse primary cardiomyocytes were performed according to our previously published methods [43]. To recapitulate the effects of septic cardiomyopathy in a cell culture setting, we exposed cardiomyocytes and HL-1 cells to a proinflammatory stimulus by treating them with 10 μg/ml LPS from Sigma-Aldrich for a duration of 72 h. Furthermore, to suppress FUNDC1 expression, HL-1 cells underwent transfection with shRNA under LPS stimulation, employing Lipofectamine 3000 (Invitrogen, Carlsbad, CA, USA). HL-1 cells were treated with 25 ng/ml rFGF2 12 h prior to LPS stimulation.

### Determination of mitochondrial membrane potential and mitophagy activity

JC-1 staining was used to determine mitochondrial membrane potential [44]. Mitophagy activity was determined via the co-immunofluorescence of mitochondria and lysosomes in HL-1 cells according to a previous study [45]. We employed a lysosome-specific staining solution (Abcam, USA) to visualize lysosomal structures and a mitochondria-specific fluorescent probe, MitoFluor (Molecular Probes, USA), to label mitochondrial organelles, enabling the detection and analysis of these subcellular compartments.

### Cell viability measurement

LDH release, an indicator of cell membrane integrity and cytotoxicity, was measured using the LDH Assay Kit (ab65393, Abcam, Cambridge, UK) following the manufacturer’s protocol [46]. The MTT and CCK-8 assays were conducted as previously described [43].

### Immunofluorescence staining

For immunofluorescence analysis, samples were prepared using a solution containing 4% paraformaldehyde, 0.1% Triton X-100 in PBS, and 1% bovine serum albumin. The specimens were then incubated overnight at 4 °C with primary antibodies targeting Gr-1 (Abcam, #ab314120) and Tn-T (Abcam, #ab8295), followed by application of appropriate secondary antibodies. To assess cellular apoptosis, TUNEL staining was performed using the Click-iT Plus TUNEL Assay Kit for In Situ Apoptosis Detection (C1088, Beyotime), adhering to the manufacturer’s protocol. Additionally, DHE staining (Invitrogen, San Diego, CA, USA) was employed to evaluate oxidative stress. Cell nuclei were counterstained with 4′,6-diamidino-2-phenylindole (DAPI). Fluorescence imaging was conducted using an Olympus fluorescence microscope to visualize and analyze the prepared samples.

### Echocardiography

Echocardiography was performed on mice by proficient researchers to observe changes in cardiac function, including wall thickness, cardiac output, and ejection fraction [47]. Myocardial contractile and diastolic functions were measured using Doppler flow imaging, indicated by PS, TR90, and TPS [48].

### Real-time qPCR

Cardiomyocyte RNA was isolated using TRIzol reagent (Invitrogen) and subsequently converted to cDNA employing the SuperScript IV First-Strand Synthesis System (Invitrogen), following the manufacturer’s guidelines. Quantitative real-time PCR was then conducted as per the provided protocol. The PCR thermal cycling parameters were established as follows: an initial denaturation step at 95 °C for 30 s, followed by 40 cycles of denaturation at 90 °C for 10 s, annealing at 60 °C for 30 s, and extension at 72 °C for 50 s. Glyceraldehyde-3-phosphate dehydrogenase (GAPDH) primers served as the internal control for relative gene expression analysis. Data analysis was performed using LightCycler 96 SW 1.1 software to interpret the qPCR results and quantify gene expression levels [49].

### Dimension reduction, clustering, and differentially expressed gene analysis

Single-cell analysis data (GSE190856) from mouse myocardial tissue, with and without septic stress, were downloaded and processed according to the methodology described in study [21]. We quantified key parameters such as the number of molecules per cell (nCount RNA) and the number of detected genes per cell (nFeature RNA), which were compared with sequencing read counts to verify the quality of the data. We used the unified manifold approximation and projection (UMAP) for dimensionality reduction to perform cell clustering after principal components were filtered, facilitating the distinct visual grouping of cell clusters. Statistically significant cell marker genes (adjusted *P* values < 0.05) were pinpointed and utilized for identifying the cell clusters’ class groups. This process involved comparing cell marker genes from the DISCO database (https://www.immunesinglecell.org/) with class-specific genes, which helped elucidate the distribution and prevalence of central genes within various cell subpopulations.

### Proteomic analysis via mass spectrometry and functional enrichment analysis

Following treatment, heart tissue samples were preserved at −80 °C for subsequent analysis by mass spectrometry. Proteins were then extracted and prepared for proteomic studies according to established protocols [50]. To elucidate the functional roles of identified targets, we performed comprehensive functional enrichment analyses, including Gene Ontology (GO) categorization, which encompassed molecular functions, biological processes, and cellular components, to annotate the genes. Additionally, we conducted KEGG analysis to correlate gene functions with genomic data, providing insights into the functions of septic cardiomyopathy-associated target genes. For these analyses, we utilized the “cluster Profiler” and “GO plot” tools (version 1.0.2), which are widely recognized for their robustness in functional analysis.

### GSEA analysis

To identify differentially expressed genes, we generated a comprehensive list of genes, ranked by their log_2_ fold-change values. We then performed GSEA using the cluster Profiler package in R/Bioconductor, which calculates an enrichment score (ES) based on the weighted Kolmogorov–Smirnov statistic. The ES was normalized to account for gene set size, and statistical significance was determined using a false discovery rate (FDR) threshold of 0.05, thereby ensuring the robust identification of enriched gene sets.

### ATP and mROS determination

The CellTiter-Glo Luminescent Viability Assay (Promega, Madison, WI, USA) was used to measure ATP concentration. The CellTiter-Glo reagent was added to the cell mixture, and the incubation system was mixed and incubated for 10 min to ensure sufficient luminescence [51]. A Tecan Infinite 200 PRO plate reader (Männedorf, Switzerland) and a cell-permeant MitoSOX Red mitochondrial superoxide indicator (Molecular Probes, USA) were used to identify luminescent signals and measure the concentration of mitochondrial ROS in the cells [52].

### ELISA assay

The concentrations of LDH, Tn-T, and CK-MB were determined using ELISA-based assays using kits from InvivoGen (San Diego, CA, USA). ELISAs from MyBioSource Inc. were applied to observe the changes of mitochondrial respiratory complex I activities (Mouse Mitochondrial Respiratory Chain Complex I ELISA Kit, ab109721, Abcam), mitochondrial respiratory complex II activities (Mouse Mitochondrial Respiratory Chain Complex II ELISA Kit, Ab109908, Abcam), and mitochondrial respiratory complex IV (Mouse Mitochondrial Respiratory Chain Complex III ELISA Kit, KTB1870, Abbkine). Luminescent signals from these assays were detected using a Tecan Infinite 200 PRO plate reader (Männedorf, Switzerland), in line with established protocols [53].

### Western blotting

Sample tissues and cells were subjected to lysis using radioimmunoprecipitation assay (RIPA) buffer, followed by protein separation via sodium dodecyl sulfate–polyacrylamide gel electrophoresis (SDS-PAGE) (acrylamide concentrations: 8%, 10%, or 12%) in a 1× running buffer (25 mM tris, 192 mM glycine, 1% SDS). The separated proteins were then transferred to polyvinylidene difluoride membranes using 1× transfer buffer (25 mM tris, 192 mM glycine, 20% methanol). The membranes were incubated with primary antibodies according to the manufacturers’ instructions, followed by detection with horseradish peroxidase-conjugated secondary antibodies. The Immobilon Western Chemiluminescent Horseradish Peroxidase Substrate (Millipore Sigma) was used for visualization, with signal detection facilitated by an enhanced chemiluminescence (ECL) substrate kit from Thermo (USA). Finally, ImageJ software (Bio-Rad, USA) was employed for image acquisition and band quantification.

### Transmission electron microscope

To prepare specimens for electron microscopy analysis, a dehydration process was conducted using a combination of acetonitrile and increasing concentrations of methanol. The samples were then embedded in EMbed-812, an epoxy resin obtained from Electron Microscopy Sciences in Hatfield, Pennsylvania. The embedded samples underwent polymerization by incubation at 70 °C for a minimum of 12 h. Subsequently, imaging was performed using a Hitachi H600 Electron Microscope, manufactured by Hitachi in Tokyo, Japan [54].

### Statistics

The data of the study were analyzed using one-way analysis of variance (ANOVA) and presented as mean ± standard error of the mean (SEM). All sample sets were normally distributed as tested with the Shapiro–Wilk normality test. Two-tailed unpaired Student’s *t* test was used to compare 2 groups; one-way ANOVA followed by Dunnett’s multiple comparisons test was used to compare differences when multiple groups were compared with a single control; one-way ANOVA followed by Tukey’s multiple comparisons test was used to compare differences when multiple groups were compared with each other; 2-way ANOVA followed by Tukey’s multiple comparisons test was used for comparisons when there were 2 independent variables. Statistical Package for the Social Sciences (SPSS Inc., Chicago, IL, USA) software, version 20.0, was used to analyze the data, and statistical significance was set at *P* < 0.05.

## Data Availability

The original data presented in the study are included in the article; further inquiries can be directed to the corresponding author/s.
