# Supplementary material for: FGF2 Attenuated Inflammation-Mediated Cardiac Damage: A Novel Mechanistic Insight into the AMPK–FUNDC1–Mitophagy
Source: Research (Wash D C). 2025 Nov 24;8:1013. doi: 10.34133/research.1013 (PMC12641185; doi:10.34133/research.1013)
Supplement: Supplementary 1 — Figs. S1 to S4 [file research.1013.f1.pdf]

Supplemental Figures

Supplementary Figure 1

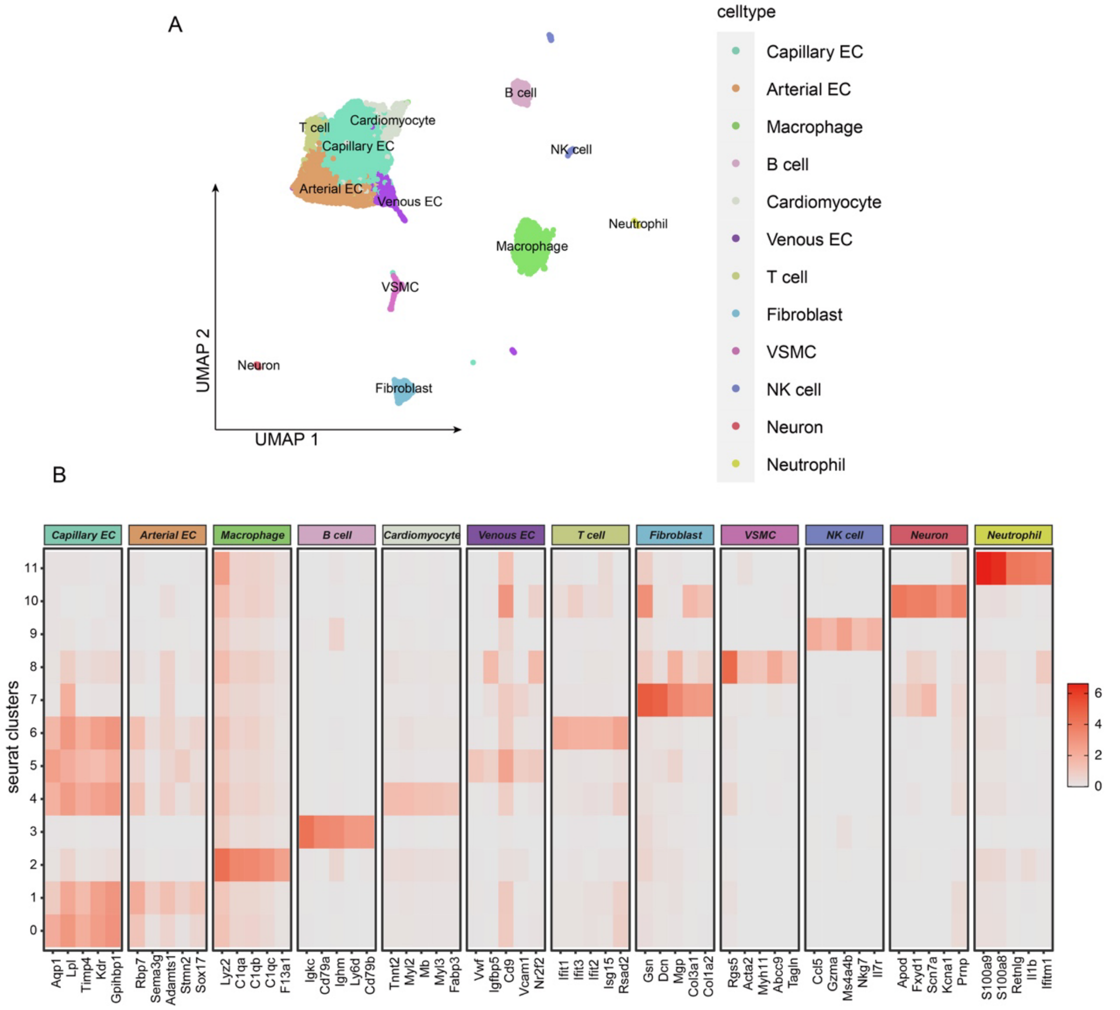

Figure S1. LPS inhibits FGF2 expression in cardiomyocyte.

(A) UMAP dimensionality reduction plots of mouse cardiac tissue cells after clustering.

(B) Cell-type-specific signature genes.

Supplementary Figure2

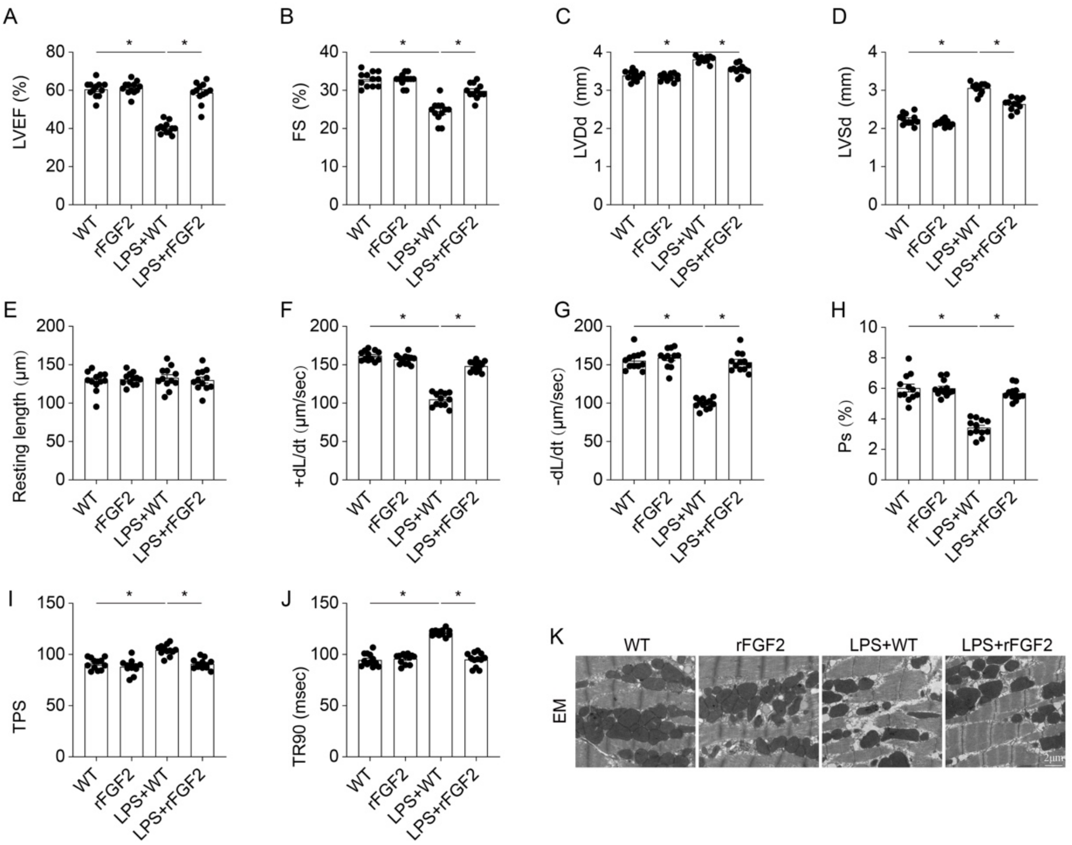

Figure S2. FGF2 improves cardiac function in sepsis-induced cardiomyopathy *in vivo* and *in vitro*.

(A–D) Cardiac function was evaluated through echocardiography. (E) Resting cell length. (F) Maximal velocity of shortening (+dL/dt). (G) Maximal velocity of relengthening (-dL/dt). (H) Peak shortening (normalized to cell length). (I) Time-to-peak shortening (TPS). (J) Time-to-90% relengthening (TR90). (K) TEM was used to observe the ultrastructural changes after LPS treatment in vivo. Data are shown as the means ± SEM (n=12/group). *$P < 0.05$.

Supplementary Figure3

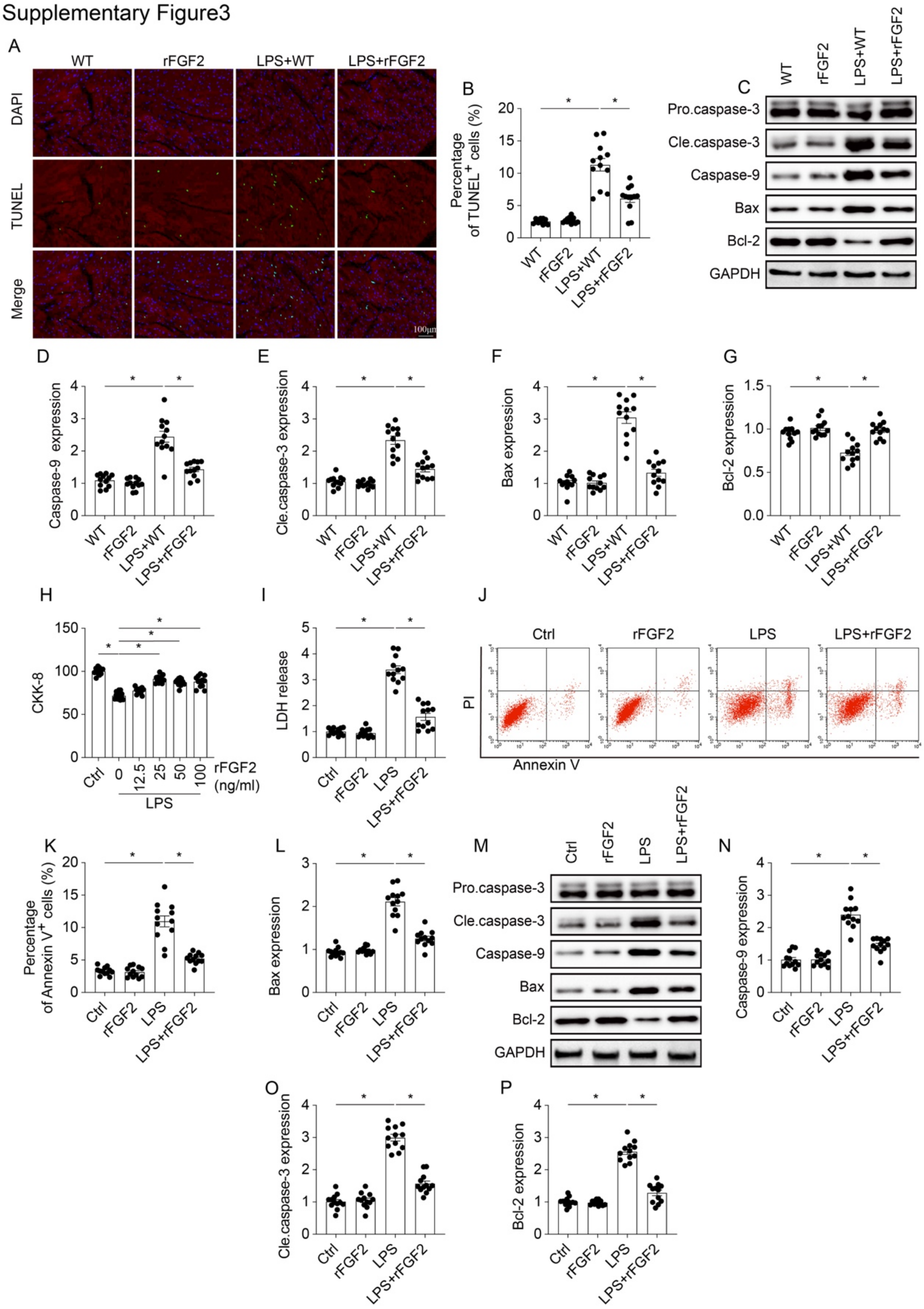

Figure S3. FGF2 preserves sepsis-induced cardiomyocyte apoptosis *in vivo* and *in intro*.

(A, B) The TUNEL assay was used to evaluate cellular apoptosis in cardiac tissue and the percentage of TUNEL-positive cells was measured. (C-G) Western blots were used to analyze the change in mitochondrial apoptotic proteins *in vivo*. (H) CCK-8 assay was performed to measure the rate of injured HL-1 cell proliferation adopted with different

concentrations of FGF2. The results indicated that treatment with 25 ng/ml FGF2 significantly increased cell viability. (I) LDH release was measured *in vitro*. (J-K) Cellular apoptosis was determined by Annexin V/PI staining in vitro. (L-P) Western blots was used to analyze the change in mitochondrial apoptotic proteins *in vitro*. Data are shown as the means ± SEM (n=12/group). *$P < 0.05$.

Supplementary Figure4

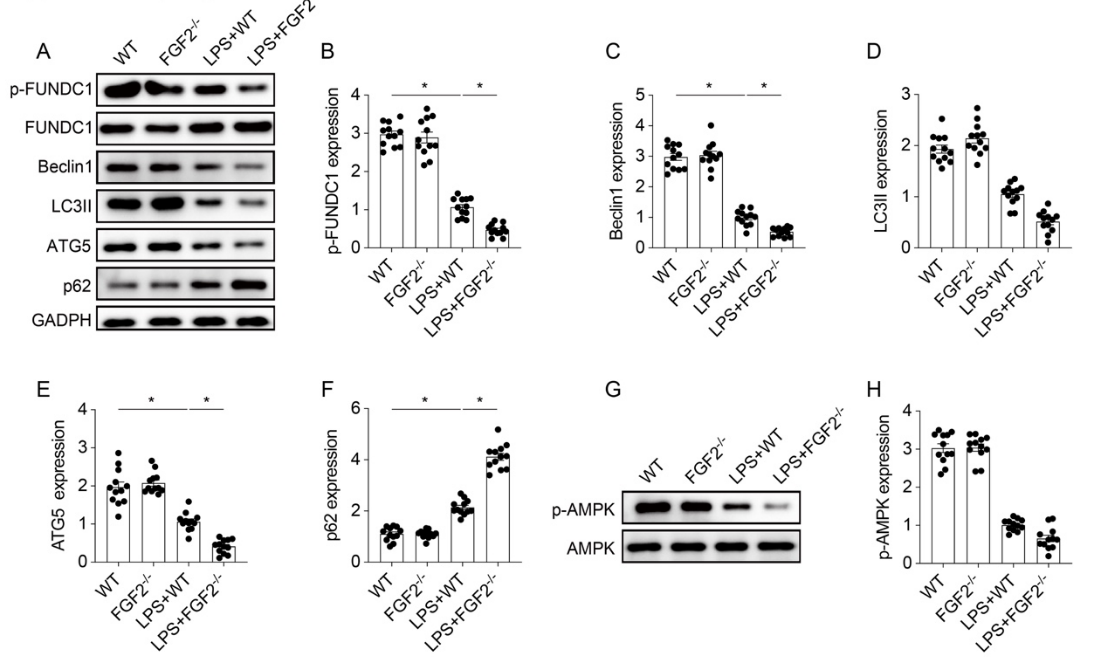

Figure S4. FGF2 deficiency exacerbates LPS-induced suppression of mitophagy and AMPK activation in cardiac tissue (n=12/group).

(A) Representative Western blots showing protein levels of p-FUNDC1, total FUNDC1, Beclin1, LC3II, ATG5, and p62 in cardiac lysates from the indicated groups. Quantification of the relative protein expression of p-FUNDC1 (B), Beclin1 (C), LC3II (D), ATG5 (E), and p62 (F) from the Western blots. G) Representative Western blot showing protein levels of p-AMPK and total AMPK. (H) Quantification of the relative protein expression of p-AMPK from the Western blots. Data are shown as the means ± SEM (n=12/group). *$P < 0.05$.
